# Solid-Phase Chemical Synthesis of Stable Isotope-Labeled RNA to Aid Structure and Dynamics Studies by NMR Spectroscopy

**DOI:** 10.3390/molecules24193476

**Published:** 2019-09-25

**Authors:** Owen Becette, Lukasz T. Olenginski, Theodore K. Dayie

**Affiliations:** Center for Biomolecular Structure and Organization, Department of Chemistry and Biochemistry, University of Maryland, College Park, MD 20742, USA; obecette@umd.edu (O.B.); lolengin@umd.edu (L.T.O.)

**Keywords:** stable isotope, phosphoramidite, solid-phase synthesis, NMR spectroscopy, RNA, structure, dynamics

## Abstract

RNA structure and dynamic studies by NMR spectroscopy suffer from chemical shift overlap and line broadening, both of which become worse as RNA size increases. Incorporation of stable isotope labels into RNA has provided several solutions to these limitations. Nevertheless, the only method to circumvent the problem of spectral overlap completely is the solid-phase chemical synthesis of RNA with labeled RNA phosphoramidites. In this review, we summarize the practical aspects of this methodology for NMR spectroscopy studies of RNA. These types of investigations lie at the intersection of chemistry and biophysics and highlight the need for collaborative efforts to tackle the integrative structural biology problems that exist in the RNA world. Finally, examples of RNA structure and dynamic studies using labeled phosphoramidites are highlighted.

## 1. Introduction

RNAs are involved in numerous cellular functions, such as gene regulation, catalysis, signaling, and retroviral infection [1,2,3,4,5,6]. RNA owes this functional diversity, in part, to its ability to change its structure on a wide range of timescales [7,8,9,10]. Therefore, biophysical techniques that characterize RNA structure and dynamics at atomic-resolution are needed to fully elucidate their function. Although X-ray crystallography and cryo-electron microscopy (cryo-EM) provide insight into RNA structure, nuclear magnetic resonance (NMR) spectroscopy is the only high-resolution structural technique capable of probing RNA dynamics on functionally relevant timescales in solution [7,8,9,11,12,13]. However, NMR studies of RNA face two challenges: narrow chemical shift dispersion and broad linewidths, both of which worsen in large RNAs [14,15,16]. A widely-used method to address these challenges is the selective incorporation of stable isotope labels such as fluorine-19 (^19^F), carbon-13 (^13^C), hydrogen-2 (^2^H), and nitrogen-15 (^15^N) into RNA [15,17,18,19,20,21,22,23,24,25,26,27,28,29]. 

Isotope labeled RNAs are predominantly prepared by in vitro transcription with T7 RNA polymerase (T7 RNAP) using well established protocols [30,31]. This approach is applicable to large RNA sequences and allows for uniform or nucleotide-specific labeling by mixing labeled with unlabeled ribonucleoside-5′-triphosphates (rNTPs). Although this method produces pure material in quantities amenable to NMR analysis (~0.1–1 μmol), signal overlap presents a significant problem for large RNAs. Moreover, T7 RNAP requires one or more guanosine (G) or adenosine (A) residues at the 5′-end for efficient in vitro RNA transcription [30,31,32]. In some cases, the addition of the 5′-G overhang(s) can alter the function of the RNA [33]. Additionally, the first 3 nucleotides (nts) at the transcription start site have a significant effect on the 5′-heterogeneity of the RNA transcripts and care must be taken when choosing these sequences [34]. To overcome the non-specificity of T7 RNAP-based in vitro transcription, three methods to prepare position-specifically labeled RNAs have emerged. In the first, Keyhani et al. developed a chemo-enzymatic synthesis that involves a single incorporation of a modified nucleoside-3′,5′-bisphosphate to the 3′-end of an RNA fragment followed by DNA-splinted ligation to complete the desired internally labeled RNA sequence [19]. This method was used to introduce photocaged, photoswitchable, and isotopically labeled nucleosides into RNAs up to 392 nts. Furthermore, this method uses standard laboratory equipment and commercially available enzymes T4 RNA ligase 1, shrimp alkaline phosphatase, and T4 RNA ligase 2, making it readily accessible to most biochemical/biophysical research groups. However, the relatively low yields from both bis-phosphorylation of the modified nucleosides (6%–22%) and DNA-splinted ligation of the RNA fragments (9%–49%) are a major drawback of this approach. 

Secondly, Liu et al. developed a hybrid solid-liquid phase transcription technique using an automated robotic platform known as PLOR (position selective labeling of RNA) to prepare isotopically labeled RNAs up to 104 nts and fluorescently and heavy atom labeled RNAs up to 71 nts [35,36,37]. In this approach, the DNA template is attached to a bead and transcription is initiated by the addition of T7 RNAP and a mixture missing one (or more) of the four rNTPs, causing RNA elongation to stall at the first position where the missing rNTP would be incorporated. The beads are then washed to remove unincorporated rNTPs and T7 RNAP. Elongation is then resumed by the addition of a new mixture containing the previously omitted rNTP. Repetition of the elongation, stalling and reinitiation steps enables synthesis of position-selectively labeled RNA. The main drawbacks of this method are the inaccessibility of the automated synthesizer and the need for large, stoichiometric amounts of T7 RNAP and DNA template. Additionally, a nucleotide within a stretch of the same residues (e.g., an A in a GAAA tetraloop) cannot be individually labeled with PLOR. Lastly, at least the first ~10 nts of the RNA sequence must be composed of only 3 of the 4 residues to ensure proper formation of the processive elongation complex and subsequently stall it.

**Figure 1:**
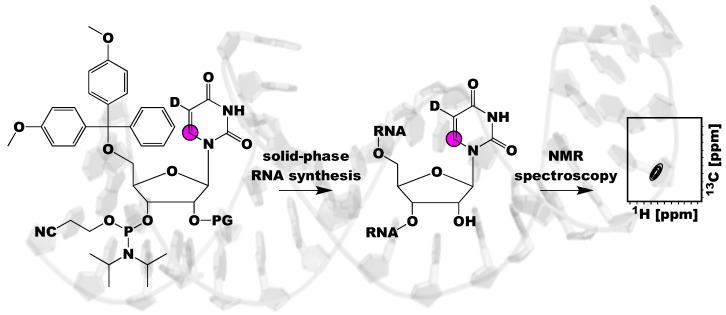
Schematic of the utility of using isotope labeled RNA phosphoramidites and solid-phase synthesis to investigate RNA structure and dynamics. This methodology incorporates labels site-specifically into RNA, resolving NMR spectral overlap and ambiguities in NMR spectra interpretation. Magenta circle indicates a ^13^C atom, D indicates a ^2^H atom, and PG refers to a 2′-OH protecting group.

The final method, solid-phase chemical synthesis of RNAs with nucleoside phosphoramidites, is the main focus of this review. This approach allows for position-specific labeling that greatly reduces NMR spectral complexity and simplifies resonance assignment (Figure 1). Furthermore, solid-phase synthesis has no sequence requirements, unlike T7 RNAP and PLOR. Despite these advantages, solid-phase synthesis is currently limited to labeling RNAs less than 76 nts [38]. Fortunately, many biologically relevant RNAs fall within this size limit [6]. For RNAs larger than 76 nts, ligation of smaller RNA strands is required [39]. As will be described, the most effective RNA labeling strategies require a combination of solid-phase and chemo-enzymatic synthesis, RNA ligation, and T7-RNAP-based transcription.

## 2. Solid-Phase Chemical RNA Synthesis

### 2.1. The Phosphoramidite Method

**Figure 2:**
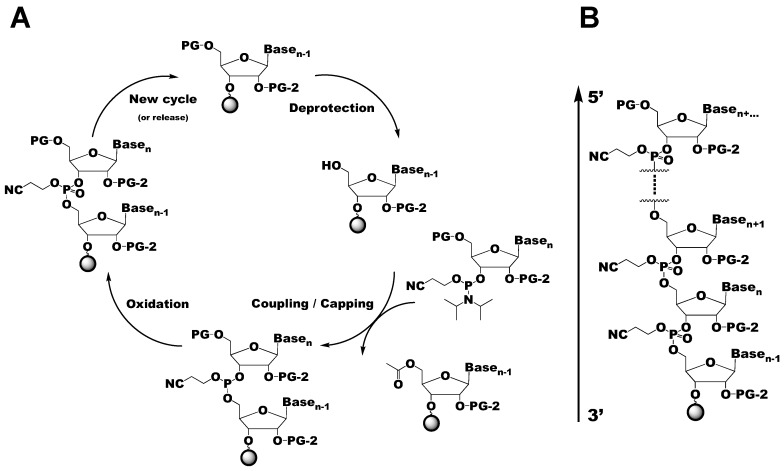
Overview of the solid-phase chemical RNA synthesis cycle. (**A**) Schematic of the solid-phase synthesis cycle of RNA, with the coupling of residue n-1 and n shown. PG refers to the 5′-OH protecting group, which is generally DMTr. PG-2 refers to the 2′-OH protecting group, which comes in many varieties, as discussed in this review. Additional cycles can be added to the synthetic cycle to grow the RNA polymer from 3′–5′ (**B**).

The solid-phase chemical synthesis of RNA via the phosphoramidite method is a four-step process that is carried out in an automated synthesizer (Figure 2A) [40,41]. Although originally developed for DNA synthesis by Beaucage and Caruthers, the phosphoramidite method has since been adapted for RNA synthesis [42]. This strategy utilizes the reaction between an activated nucleoside phosphoramidite and a solid-support bound nucleoside to synthesize the desired RNA sequence in the 3′ to 5′ direction (Figure 2B). During the first step, the 4,4′-dimethoxytrityl (DMTr) protecting group at the 5′-hydroxyl (OH) of the tethered 3′-nucleoside is removed with trichloroacetic acid or dichloroacetic acid. The nucleophilic 5′-OH then attacks and removes the activated phosphoramidite to couple the two nucleosides with a phosphite triester bond. To minimize the amount of undesired RNA sequences, the unreacted tethered 3′-nucleosides are capped by acetylation of the 5′-OH groups. The final step is the oxidation of the phosphite triester to a phosphotriester with either iodine (I_2_) or tert-butylhydroperoxide. After the entire sequence has been synthesized, the RNA is deprotected and cleaved from the solid-support. This strategy provides a powerful alternative to other methods of producing synthetic RNAs such as enzymatic ligation [43,44,45,46].

**Figure 3:**
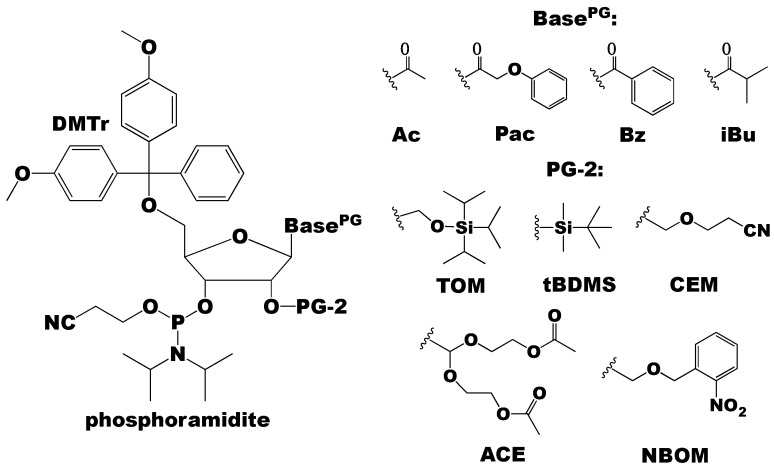
The phosphoramidite building blocks can have different nucleobase (exocyclic amino) and 2′-OH protecting groups (PG and PG-2, respectively). Common nucleobase protecting groups are acetyl (Ac), phenoxyacetyl (Pac), benzoyl (Bz), and isobutyryl (iBu). The different 2′-OH protecting groups are discussed throughout this review, but common groups include [(triisopropylsilyl)oxy]methyl (TOM), tert-butyldimethylsilyl (tBDMS), 2′-cyanoethoxymethyl (CEM), bis(acetoxyethoxy)methyl ether (ACE), and photolabile 2-nitrobenzyloxymethyl (NBOM).

The efficiency of the chemical synthesis of RNA by the phosphoramidite method depends on the protecting groups used, specifically at the exocyclic amino groups of the nucleobase (A, C, and G), and the 2′- and 5′-OH functionalities of the ribose sugar (Figure 3) [40]. Typically, the 5′-OH group is protected with DMTr and the nucleobase exocyclic amino groups are protected with acetyl (Ac), phenoxyacetyl (Pac), benzoyl (Bz), or isobutyryl (iBu) groups (Figure 3). In contrast to the 5’-OH and exocyclic amino protecting groups originally developed for DNA, the 2′-OH protecting groups are unique to RNA. The 2′-OH protecting groups must: (1) be stable under all reaction conditions (acidic conditions of de-tritylation, basic conditions of deprotection of the nucleobase and cleavage from the solid-support), (2) be quantitatively removed under mild conditions that do not degrade the RNA, and (3) not interfere with the phosphoramidite coupling reaction [40,47,48,49]. In the next section, we briefly summarize the commonly used 2′-OH protecting groups along with their comparative advantages and disadvantages. 

### 2.2. Choice of RNA Phosphoramidite 2’-OH Protecting Groups 

There is currently no consensus RNA 2′-OH protecting group, but the available groups can be broadly categorized into acid-, photo-, and fluoride-labile groups [40,50,51,52,53,54]. For generating isotope labeled RNAs, the fluoride-labile 2′-OH protecting groups *tert*-butyldimethylsilyl (tBDMS), [(triisopropylsilyl)oxy]methyl (TOM), and 2′-cyanoethoxymethyl (CEM) group are preferred. Despite the stability under acidic conditions and ease of addition and removal, tBDMS suffers from a low coupling efficiency, long coupling time, and migration from the 2′- to 3′-OH during RNA synthesis [40]. Despite its limitations, 2′-*O*-tBDMS phosphoramidites have been successfully used to synthesize labeled RNAs up to 53 nts [55]. Compared to tBDMS, TOM has shorter coupling times (~6 min) and does not migrate from 2′- to 3′-OH [40]. With TOM, labeled RNAs as large as 55 nts have been successfully prepared [56]. The CEM group has a high coupling efficiency (~99%), short coupling time (2–4 min), and minimal steric hindrance that allows synthesis of RNAs as large as 170 nts [57,58]. In terms of labeled RNA, sequences up to 76 nts have been successfully prepared for NMR analysis with 2′-*O*-CEM phosphoramidites [38]. Two other 2′-OH protecting groups that have not yet been applied to RNA labeling are the photolabile 2-nitrobenzyloxymethyl (NBOM) group and the acid-labile bis(acetoxyethoxy)methyl ether (ACE) group [47,53,54]. The NBOM group is stable under acidic and basic conditions, has a short coupling time (2 min), and high coupling efficiency. However, its removal is not quantitative [40]. While the ACE group has a short coupling time (1 min) and is capable of generating RNAs in high yield and purity, it is acid labile and therefore not compatible with 5′-DMTr deprotection. Thus, fluoride-labile 5′-OH protecting groups are required and the synthesizer has to be modified to handle triethylamine/trihydrofluoride (TEA-3HF) as a deprotection agent [40]. Nevertheless, the ACE group is commercially successful at preparing RNAs in large quantities even with modified nucleosides (e.g., inosine, *N*^6^-methyladenosine, pseudouridine, 2’-fluoro-A,U,G,C) [47,53].

Given the advantages and disadvantages of each 2′-OH protecting group, the choice of which to use typically depends on the downstream application. However, few 2′-OH protecting groups are capable of making RNA in high enough quantity and purity for NMR spectroscopy. As will be discussed, 2′-*O*-tBDMS and 2′-*O*-TOM phosphoramidites have been used to great success to study RNA structure and dynamics by NMR spectroscopy [56,59,60,61,62]. However, phosphoramidite synthesis with these protecting groups is limited to RNAs less than 60 nts. Nevertheless, unlabeled 2′-*O*-tBDMS and 2′-*O*-TOM phosphoramidites are commercially available, so researchers only need to synthesize isotope labeled phosphoramidites in-house, dramatically simplifying sample preparation. The commercially unavailable 2′-*O*-CEM is the only protecting group that can accommodate larger RNAs, but both unlabeled and labeled phosphoramidites must be synthesized in-house. In practice, 2′-*O*-tBDMS phosphoramidites are used for NMR structure and dynamics studies of RNAs prepared by solid-phase synthesis, unless the RNA under investigation is greater than 60 nts. Still, very few research groups have the resources (organic laboratory and solid-phase synthesizer) and expertise (NMR spectroscopy) necessary to fully exploit these technologies. One goal of this review is to motivate the collaboration of organic chemists with biophysicists to unlock the power of this method to study RNA structure, dynamics, and function.

## 3. Stable Isotope Labeling of RNA Phosphoramidites

In principle, any method to incorporate isotope labels into nucleobases, nucleosides, or rNTPs can be converted into phosphoramidites, using one or more of the synthetic routes described herein. The diverse methodologies to generate such labeled molecules were recently reviewed [20]. Once synthesized, phosphoramidites are characterized by ^1^H/^13^C/^31^P NMR and mass spectrometry. The latter technique is complicated by the acid-labile nature of the phosphoramidites, which requires specialized electro-spray ionization techniques in non-aqueous buffers with lithium chloride for accurate mass determination [63,64]. Several groups have developed synthetic strategies to obtain ^13^C/^15^N-labeled RNA phosphoramidites with a variety of 2′-OH protecting groups [38,39,55,56,59,60,61,62,65,66,67,68]. This section will highlight the many efforts employed to synthesize labeled RNA phosphoramidites.

Jones and colleagues developed a chemo-enzymatic synthetic strategy to prepare 2′-O-tBDMS phosphoramidites from isotope labeled nucleosides prepared by de novo biosynthesis [69,70,71,72,73,74]. Starting from labeled adenosine or guanosine, the phosphoramidites were prepared in 7 steps with overall yields of 60% [66,74]. Furthermore, 85%–90% regioselective 2′-silylation was achieved by concomitant treatment of *N*-Pac-,5′-DMTr-adenosine or -guanosine with a mixture of phenyl-*H*-phosphonate, *tert*-butyldimethylsilyl chloride (tBDMS-Cl), and 1,8-diazabicyclo[5.4.0]undec-7-ene (DBU) [66,74]. The [7,NH_2_-^15^N_2_]-adenosine was prepared in 7 steps with 45% yield starting from an inexpensive pyrimidine, 6-amino-2-thioxo-1,2-dhydro-4(3*H*)-pyrimidone, and [^15^N]-NaNO_2_ and [^15^N]-NH_4_Cl as ^15^N sources for N7 and NH_2_, respectively [69]. As an intermediate, [7-^15^N]-hypoxanthine was generated in 81% yield and is readily converted to [7-^15^N]-6-chloropurine for enzymatic transglycosylation with commercially available purine nucleoside phosphorylase (EC 2.4.2.1) and 7-methylguanosine as the ribose source. Selective amination at C6 was achieved with [^15^N]-NH_4_Cl. [8-^13^C-1,7,NH_2_-^15^N_3_]-adenosine was prepared similarly, except during the ring closure of the 4,5-diaminopyrimidine intermediate the ^13^C label was introduced by [^13^C]-sodium ethyl xanthate [73]. Either [8-^13^C-1,7,NH_2_-^15^N_3_]-adenosine or -guanosine can be synthesized starting with [8-^13^C-7,NH_2_-^15^N_2_]-adenosine in 5 steps with 60%–62% yield and 4 steps with 65%–70% yield, respectively. Although this synthetic strategy is well suited for purine nucleoside phosphoramidites, the 2′-*O*-tBDMS regioselectivity is not applicable to pyrimidines [74]. Virtually any labeled nucleobase can be readily coupled to ribose via commercially available purine nucleoside phosphorylase, as recently demonstrated [75]. Although labeled nucleobase is readily coupled to ribose using phosphorylases, the introduction of labeled ribose remains a challenge.

Wenter and Pitsch developed a convenient approach to obtain [1-^15^N]-purines and [3-^15^N]-pyrimidines by N1/N3 nitration of unlabeled protected nucleoside precursors and subsequent substitution with in situ generated ^15^NH_3_ [56,65,76]. Wenter and Pitsch synthesized [1-^15^N]-adenosine and -guanosine and [3-^15^N]-uridine and -cytidine 2′-*O*-TOM phosphoramidites starting from commercially available unlabeled *N^6^*-Ac,5′-*O*-DMTr,2′-*O*-TOM-adenosine and 5′-*O*-DMTr,2′-*O*-TOM-uridine, respectively, with [^15^N]-NH_4_Cl as the ^15^N source [65]. In this approach, [1-^15^N]-adenosine was prepared in 5 steps with 37% yield and an inosine intermediate was used as a starting point to synthesize [1-^15^N]-guanosine in 6 steps with 24% yield. [3-^15^N]-uridine was obtained in 3 steps with 49% yield and was converted to [3-^15^N]-cytidine with 81% yield in two additional steps. The isotope labeled, *N*-Ac,5′-*O*-DMTr,2′-*O*-TOM-nucleosides were then converted into phosphoramidites with 2-cyanoethyl diisopropylphosphoramidochloridite (CEP-Cl) in 1 step with at least 90% yield. The main advantage of this synthesis is that the incorporation of ^15^N occurs after the low yielding 2′-*O*-TOM protection.

Building on the work of Wenter and Pitsch, Neuner et al. prepared [1-^15^N]-purine and [3-^15^N]-pyrimidine phosphoramidites with either 2′-*O*-tBDMS or 2′-*O*-TOM protection [56]. Neuner et al. started their purine and pyrimidine syntheses with a tri-acetylated inosine and tri-tBDMS uridine, respectively. From the inosine precursor, the [1-^15^N]-adenosine phosphoramidite was prepared in 8 steps with 16% yield. During this synthesis, a nitroso-inosine intermediate was used to synthesize the [1-^15^N]-guanosine phosphoramidite in 11 steps and 9% yield. As far as the pyrimidines were concerned, [3-^15^N]-uridine was synthesized starting from unlabeled 2′-*O*-tBDMS-uridine in 6 steps with 22% yield. The [3-^15^N]-cytidine was obtained from 2′-*O*-tBDMS-[3-^15^N]-uridine in 5 steps with 25% yield and [3,NH_2_-^15^N_2_]-cytidine was synthesized in 6 steps with 16% yield. This protocol circumvents the need for the expensive starting materials used by Wenter and Pitsch but at the expense of lower reaction yields [65].

From isotope labeled nucleobases [25,26,27,38,55,60,77], the groups of Kreutz and Micura prepared 2′-*O*-tBDMS and 2′-*O*-TOM phosphoramidites for NMR studies [59,60,61,62]. For example, uracil was synthesized, following procedures developed by Santa Lucia and Tinoco [77]. First, ^13^C was installed at C6 from [^13^C]-KCN, C5 from [2-^13^C]-bromoacetic acid, C4 from [1-^13^C]-bromoacetic acid, C2 from [^13^C]-urea, and ^15^N was inserted at N1 and N3 with [^15^N]-urea [77]. The labeled uracil was then coupled to 1′-*O*-acetyl-(2′,3′,5′-*O*-tribenzoyl)-β-d-ribofuranose under Vorbrüggen conditions using Lewis acids such as trimethylsilyl trifluoromethanesulfonate (TMSOTf) as a selective catalyst [59,60]. The ribose-protected uridine nucleoside was fully deprotected and the 5′-OH was DMTr protected with 60% yield. From the 5′-*O*-DMTr-uridine, either the 2′-*O*-tBDMS or 2′-*O*-TOM phosphoramidites were obtained in 2 steps with 46% or 26% yield, respectively [59,60]. The 2′-*O*-TOM-cytidine phosphoramidite was readily accessible from the 5′-*O*-DMTr,2′-*O*-TOM-uridine intermediate in 4 steps with 45% yield. The ^13^C-purine phosphoramidites were synthesized from morpholine using [^13^C]-thiourea and [^13^C]-formic acid to install ^13^C at adenine C2 and purine C8, respectively [27,55,77].

Finally, Kremser et al. synthesized isotope labeled 2′-*O*-CEM phosphoramidites using a protocol similar to Ohgi et al., using sequential 3′,5′-*O*-(tetraisopropyldisiloxane-1,3-diyl) (TIPDS)-protection, 2′-*O*-CEM protection, 3′,5′-*O*-TIPDS deprotection, 5′-O-DMTr protection, and phosphoramidite conversion [38,48,52]. Kremser et al. prepared [8-^13^C]-adenosine and -guanosine and [6-^13^C-5-^2^H]-uridine and -cytidine 2′-*O*-CEM phosphoramidites [38]. Starting from 3′,5′-*O*-TIPDS-adenosine, [8-^13^C]-adenosine was synthesized in 5 steps with 29% yield. The [8-^13^C]-guanosine 2′-*O*-CEM phosphoramidite was synthesized de novo from the labeled nucleobase in 9 steps with 14% yield. Synthesis of [6-^13^C-5-^2^H]-uridine 2′-O-CEM phosphoramidite began from the unprotected labeled nucleoside, and proceeded to the phosphoramidite in 5 steps with 13% yield. Finally, [6-^13^C-5-^2^H]-cytidine 2′-O-CEM phosphoramidite was synthesized from 3′,5′-*O*-TIPDS-uridine in 6 steps with 44% yield. The major disadvantage of 2′-*O*-CEM phosphoramidites is the need to synthesize both unlabeled and stable isotope-labeled material since they are not commercially available. There are also more synthesis steps compared to tBDMS and TOM labeling protocols. Taken together, many stable isotope labeled RNA phosphoramidites have been synthesized with diverse labeling patterns. The next section is devoted to highlighting a few examples of how these labels can be exploited to study RNA structure and dynamics by NMR spectroscopy.

## 4. Applications to NMR Spectroscopy

### 4.1. Facile RNA Resonance Assignments and Base Pair Interactions

An unparalleled advantage of phosphoramidite solid-phase chemical synthesis over in vitro transcription is the ability to make RNA with position-specific isotope labels that dramatically simplifies unambiguous resonance assignments (Figure 1). For example, a 40 nt RNA can be assigned with 40 samples, each containing one of the 40 residues labeled. While assigning all RNA resonances with this methodology is too cumbersome for most groups, phosphoramidites are a great tool to confirm assignments from orthogonal NMR experiments or to resolve overlapped peaks in a given NMR spectra. In addition to assigning RNA resonances, various research groups have utilized ^13^C/^15^N-labeled phosphoramidites to monitor both Watson-Crick and non-canonical base pairs in RNA [56,62].

Incorporation of ^15^N-labels into RNA provides information on both canonical Watson-Crick and non-canonical base pairs in RNA [56,62,78,79,80]. [1,NH_2_-^15^N_2_]- and [2-^13^C-1,NH_2_-^15^N_2_]-guanosine were position-specifically incorporated into short (8–12 nts) RNA fragments to compare spectral properties of GU to GC base pairs [79]. Similarly, [2-^13^C-1,NH_2_-^15^N_2_]-guanosine and [7,NH_2_-^15^N_2_]-adenosine were position-specifically incorporated into short (8 nts) RNAs to study face-to-face and sheared GA base pairs [78]. Additionally, [2-^13^C-1,7,NH_2_-^15^N_3_]-guanosine and [8-^13^C-1,7,NH_2_-^15^N_3_]-adenosine were incorporated into loop A and B domains (14–24 nts) from the hairpin ribozyme found in the satellite RNA of tobacco ringspot virus [80]. In these examples, the ^13^C isotopes were used as tags to distinguish between the residues in doubly labeled samples since ^1^J_H2C2_ or ^1^J_H8C8_ splits the ^1^H resonances for residues that contain ^13^C directly coupled to ^15^N. Wenter and Pitsch incorporated [1-^15^N]-adenosine and -guanosine and [3-^15^N]-uridine and -cytidine labels into 5 sites of the 32 nt bistable RNA [65]. An HNN correlation spectroscopy (COSY) experiment was used to correlate the hydrogen bond donor and acceptor N-atoms within a base pair formed by two ^15^N-labeled residues [65,81,82]. With this approach, Wenter and Pitsch were able to unambiguously assign each HNN correlation to a particular base pair within one of the two co-existing conformers [65].

**Figure 4:**
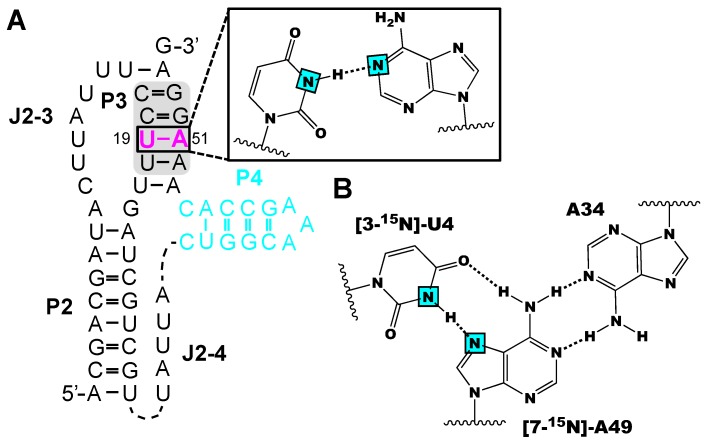
Example of NMR structural analysis of RNAs made from isotope labeled phosphoramidites. (**A**) [3-^15^N]-uridine and [1-^15^N]-adenosine labeled preQ1 (class-II) RNA facilitated the probing of the 19U-A51 base pair by HNN COSY experiments [56] (**B**) [3-^15^N]-uridine and [7-^15^N]-adenosine labeling has the potential to identify base triples in RNAs [62]. Magenta colors indicate labeled residues and the cyan squares refer to ^15^N atoms.

Neuner et al. probed the conformational flexibility of the P3 stem of PreQ1 class-II riboswitch interaction with a single-labeled ^15^N(3)-U:^15^N(1)-A base pair using their [3-^15^N]-uridine and [1-^15^N]-adenosine labels [56]. Base pair formation for different conformers were unequivocally observed based on HNN-COSY experiments, suggesting that the P3 stem is closed in the ligand-bound and unbound RNA (Figure 4A). Secondly, Neuner et al. synthesized [7-^15^N]-adenosine phosphoramidite to verify the formation of a base triplet near the active site of the env22 twister ribozyme [62]. The A49-N(7)HN(3)-U4 hydrogen-bond interaction was directly monitored with HNN-COSY experiments using chemically synthesized twister ribozyme RNA with [3-^15^N]-uridine and [7-^15^N]-adenosine incorporated at U4 and A49, respectively (Figure 4B). Unfortunately, a correlation signal between H-^15^N(3)-U4 and ^15^N(7)-A49 was lacking in the HNN-COSY spectra. Nevertheless, the use of [7-^15^N]-adenosine in HNN-COSY experiments is a straightforward way to demonstrate non-canonical base pairing interactions in RNA.

To date, two high-resolution NMR structures have been solved using position-specific labeled RNAs by solid-phase synthesis [83,84]. Traditional NMR assignment strategies are optimized for largely helical RNAs and often fail for non-canonical tertiary structural regions. Wolter et al. solved the structure of a 34-nt GTP-binding RNA aptamer that forms exotic structural features including a trans A:A base pair, a novel GACG quartet, a protonated adenine, and a GCA base triplet [83]. To assign the resonances from these unique regions, 10 site-specifically [6-^13^C]-uridine and -cytidine and [8-^13^C]-adenosine and -guanosine samples were prepared by solid-phase synthesis. A similar strategy was applied by Weickhmann et al. to solve the structure of the 43 nt SAM/SAH-binding riboswitch bound to SAH [84]. The riboswitch adopts an H-type pseudoknot characterized by extensive base pairing between bulge residues and a flexible 3′ tail. To assign residues within this region, 15 site-specifically [1,3-^15^N_2_]- uridine, [6-^13^C]-uridine and [6-^13^C]-cytidine and [2,8-^13^C_2_]-, [8-^13^C]-adenosine and [8-^13^C]-guanosine RNA samples were chemically synthesized [84,85]. The use of position-specific labeling was essential for the assignments and subsequent structure determinations of both the GTP-binding RNA aptamer and the SAM/SAH-binding riboswitches.

### 4.2. Incorporating Isolated Spin-Pairs for Artifact Free RNA Dynamic Probing

**Figure 5:**
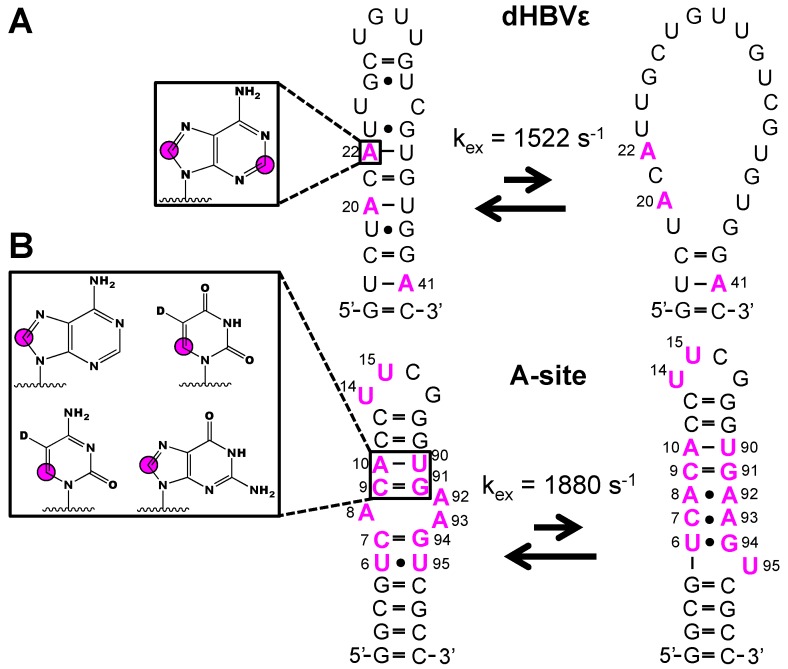
Example of dynamic probing of RNAs made from isotope labeled phosphoramidites. (**A**) Incorporation of [2,8-^13^C_2_]-adenosine into dHBVε RNA enabled ^13^C CPMG investigation of residues A20, A22, and A41 and the overall kinetic rate of unfolding to be quantified [55]. (**B**) Incorporation of [8-^13^C]-adenosine and –guanosine and [6-^13^C-5-^2^H]-uridine and –cytidine into A-site RNA allowed for the use of ^1^H CPMG studies and revealed the overall exchange rate with a minor populated state [61]. Magenta colors indicate labeled residues and magenta circles refer to ^13^C atoms.

In addition to NMR structural experiments, isotope labeled phosphoramidites have been used with great success to study RNA dynamics [39,55,59,60,61]. The isolated ^1^H-^13^C spin-pair of the [6-^13^C]-pyrimidine and [8-^13^C]-purine phosphoramidites remove the ^13^C-^13^C scalar and dipolar coupling interactions and thus facilitate straightforward probing of functional dynamics in RNA. Strebitzer and Nußbaumer et al. demonstrated the potential of atom-specific ^13^C-labeling to probe conformational dynamics in the ε-element of the duck Hepatitis B virus RNA (dHBVε) [55]. Wijmenga and co-workers previously showed that an RNA construct that represented the upper stem undergoes conformational fluctuations from the fast (pico- and nanosecond) to intermediate (microsecond) timescale [86]. To cross-validate this previous study, a dHBVε construct was made with [2,8-^13^C_2_]-adenosine incorporated at residues A20, A22, and A41. The kinetics of the unfolding process (k_ex_ = 1522 ± 128 s^-1^) were quantified via a ^13^C-CPMG relaxation dispersion (RD) experiment utilizing the ^13^C-adenosine labels (Figure 5A). In addition to ^13^C RD experiments, the absence of nearby protons makes purine ^1^H8-^13^C8 and pyrimidine ^1^H6-^13^C6 spin-pairs well suited for ^1^H RD experiments. In the latter case, pyrimidine ^1^H5 must be selectively deuterated. With this in mind, Juen et al. probed the milli- to microsecond dynamics in the 27 nt A-site RNA with ^1^H RD experiments, owing to the incorporation of [6-^13^C-5-^2^H]-uridine and -cytidine and [8-^13^C]-adenosine and -guanosine labels at various sites (Figure 5B) [61]. The overall exchange rate constant (k_ex_ = 1880 ± 140 s^−1^) between the major and minor RNA states was obtained from the ^1^H RD profiles of C7, C9, A10, A93, G94, and U95. Moreover, these data agreed with the previously reported ^13^C RD experiments, validating the ^1^H RD approach to study milli- to microsecond dynamics in RNA. These approaches are limited to RNAs less than 70 nts and only labeled nucleobase is readily coupled to unlabeled ribose. The introduction of labeled ribose using this method remains a challenge.

**Figure 6:**
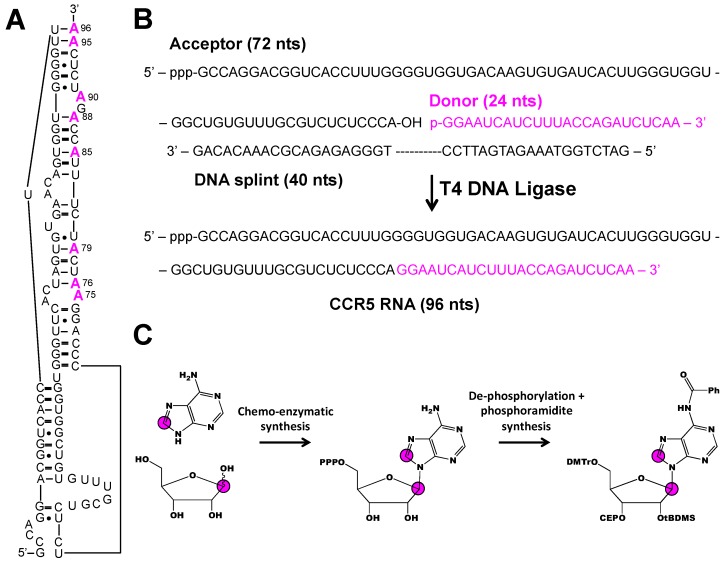
Combining chemo-enzymatic synthesis, chemical ligation, and phosphoramidite synthesis to make nucleobase and ribose isotope labeled CCR5 RNA [39]. (**A**) Secondary structure of the 96 nt CCR5 RNA. (**B**) Outline of ligation method. The unlabeled 72 nt acceptor fragment was made by in vitro transcription and the 24 nt donor fragment (colored in magenta) was made by solid-phase synthesis to incorporate individual [1′,8-^13^C_2_]-adenosine labels, as shown in (**A**). (**C**) Schematic of the combined chemo-enzymatic synthesis and phosphoramidite synthesis to build the [1′,8-^13^C_2_]-adenosine phosphoramidite. Magenta color indicates labeled residues, magenta circles refer to ^13^C atoms, and Ph refers to phenyl group.

Two strategies to overcome the inherent size limitations of solid-phase RNA synthesis involve ligation of small chemically synthesized fragments to a larger in vitro transcribed segment and use of chemo-enzymatic methodology to incorporate both isotope labeled nucleobase and ribose moieties. This approach was recently applied to interrogate the dynamics of 96 nt CCR5 RNA that stimulates -1 programmed ribosomal frameshifting upon binding to a microRNA (Figure 6A) [39]. Chen et al. divided the 96 nt construct into a 72 nt acceptor fragment and a 24 nt donor fragment (Figure 6B). Eight donor fragments, each containing a different [1′,8-^13^C_2_]-adenosine-labeled residue were prepared by solid-phase synthesis and ligated to an unlabeled acceptor fragment with T4 DNA ligase (Figure 6B,C). Not only did this approach allow for the unambiguous resonance assignment of each of the 8 A residues, it also facilitated dynamics measurements by a ^1^H1′-^13^C1′ CMPG RD experiment. Of the 7 A residues measured, 5 showed evidence of chemical exchange on the millisecond time-scale attributed to base pair opening and closing. Chen et al. identified a sparsely populated (~10%) excited state with a chemical shift matching that of the CCR5:microRNA complex. Chen et al. concluded that the CCR5 samples free and bound conformations in solution and the addition of cognate microRNA stabilizes the bound conformation.

## 5. Conclusions

**Figure 7:**
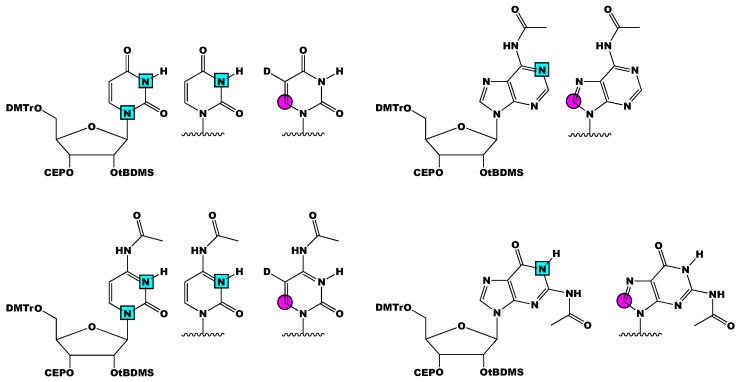
Schematic of all the isotope labeled 2′-O-tBDMS RNA phosphoramidites made commercially available by Silantes. [1,3-^15^N_2_]-, [3-^15^N]-, and [6-^13^C-5-^2^H]-uridine and [1-^15^N]- and [8-^13^C]-adenosine are shown on the top. [1,3-^15^N_2_]-, [3-^15^N]-, and [6-^13^C-5-^2^H]-cytidine and [1-^15^N]- and [8-^13^C]-guanosine are shown on the bottom. All phosphoramidites (except uridine) are *N*-Ac protected. Magenta circles represent ^13^C atoms, cyan squares represent ^15^N atoms, and D represents ^2^H atoms.

In summary, we present an overview of past and current RNA phosphoramidite isotope labeling methodologies and their applications in RNA structure and dynamic analysis by NMR spectroscopy. The biggest practical considerations in the solid-phase chemical synthesis of labeled phosphoramidites is 2′-OH protection and stable isotope labeling pattern, both of which are specified by the RNA of interest. Most NMR studies discussed herein were with RNAs prepared with 2′-*O*-tBDMS and 2′-*O*-TOM phosphoramidites, owing to the commercial availability of the unlabeled material. From the developments made by Kreutz and co-workers, Silantes has made the following labeled phosphoramidites commercially available: [3-^15^N]-, [1,3-^15^N_2_]-, and [6-^13^C-5-^2^H]-uridine and -cytidine, and [1-^15^N]-, [8-^13^C]-adenosine and -guanosine (Figure 7). The availability of ^13^C/^15^N-labeled 2′-*O*-tBDMS phosphoramidites will increase the accessibility of these methods. Thus far, analysis of RNAs greater than 60 nts is only possible with 2′-*O*-CEM phosphoramidites or enzymatic ligation of smaller RNA fragments. However, RNA structural biology is moving toward larger and larger RNAs—especially as cryo-EM gains popularity. Therefore, attention must center on: (1) synthetic approaches to incorporate isotope labels into the ribose and (2) solid-phase synthetic conditions that accommodate RNAs larger than 100 nts.
